# A Review of the Genetics of Intracranial Berry Aneurysms and Implications for Genetic Counseling

**DOI:** 10.1007/s10897-016-0029-8

**Published:** 2016-10-14

**Authors:** Emma Hitchcock, William T. Gibson

**Affiliations:** 1 Department of Medical Genetics, University of British Columbia, Vancouver, BC Canada; 2 BC Children’s Hospital, Vancouver, BC Canada

**Keywords:** Intracranial berry aneurysms, Familial intracranial aneurysms, Linkage analysis, Genome-wide association study, Whole-exome sequencing, Autosomal dominant polycystic kidney disease, Ehlers-Danlos syndrome, Marfan syndrome, Neurofibromatosis type I, Loeys-Dietz syndrome

## Abstract

Here we review the current understanding of the genetic architecture of intracranial berry aneurysms (IBA) to aid in the genetic counseling of patients at risk for this condition. The familial subtype of IBA, familial intracranial aneurysms (FIA), is associated with increased frequency of IBA, increased risk of rupture, and increased morbidity and mortality after rupture. Family history is the strongest predictor for the development of IBA. However, a genetic test is not yet available to assess risk within a family. Studies using linkage analysis, genome-wide association, and next-generation sequencing have found several candidate loci and genes associated with disease onset, but have not conclusively implicated a single gene. In addition to family history, a separate or concurrent diagnosis of autosomal dominant polycystic kidney disease is a strong genetic risk factor for IBA formation. We also discuss the relative risk for developing IBA in several Mendelian syndromes including vascular Ehlers-Danlos syndrome, Marfan syndrome, Neurofibromatosis Type I, and Loeys–Dietz syndrome.

## Introduction

**Fig. 1:**
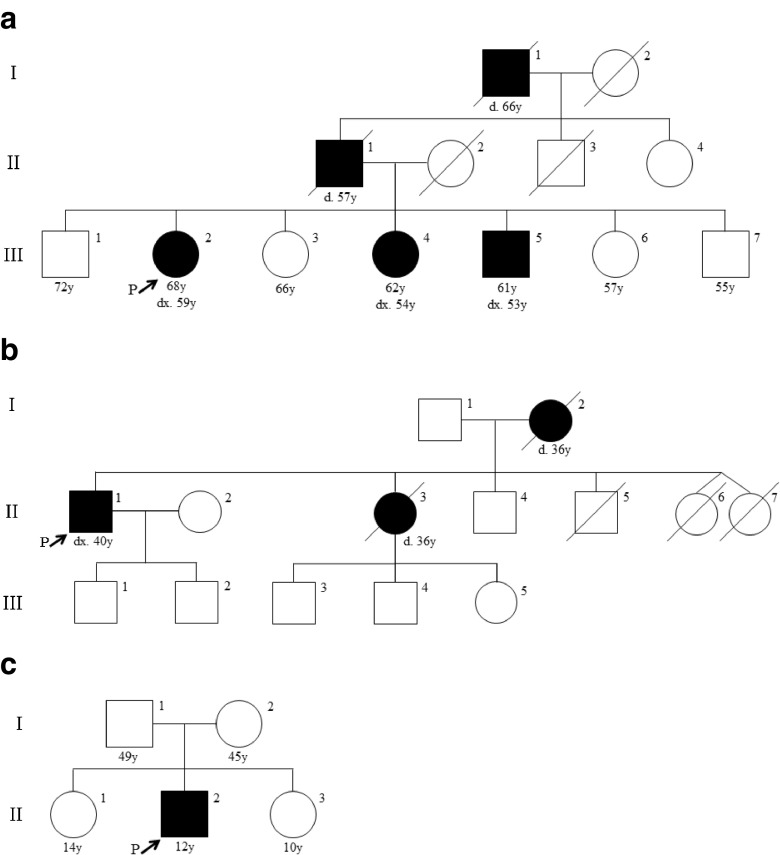
Pedigrees of three families diagnosed with familial intracranial aneurysms suggesting dominant inheritance (**a),** and inheritance consistent with dominant or X-linked transmission **(b**). Part (**c**) shows a sporadic case of an intracranial berry aneurysm; its onset in childhood in the absence of known environmental risk factors is consistent with a major genetic risk factor such as a de novo pathogenic variant

Intracranial berry aneurysms (IBA) develop in the walls of cerebral arteries where the endothelial layer has weakened and formed a sac-like abnormality. IBA are also referred to as saccular aneurysms and differ from other intracranial aneurysms in shape, forming an out-pocketing off of a cerebral artery, reminiscent of a berry on a vine. Fusiform aneurysms involve dilatation of the entire vessel wall for some component of its length; in the cerebral circulation they are much rarer that berry aneurysms (Park et al. 2008). It is estimated that 3 % of the general population have at least one IBA (Rinkel et al. 1998; Vlak et al. 2011), a prevalence that increases to 3.6–6.5 % in people over 30 years of age (Krischek and Inoue 2006). Aneurysmal rupture accounts for a significant proportion (80–85 %) of subarachnoid hemorrhage (SAH) (Brown and Broderick 2014). SAH is fatal in 35–50 % of patients, and leads to permanent brain damage in 25–50 % of survivors (Rinkel and Algra 2011; Ronkainen et al. 1998; Rosenorn et al. 1987; Schievink 1997). Population-wide screening is not currently recommended, as most cerebral aneurysms are asymptomatic and will never rupture (Ronkainen et al. 1998). From a genetic epidemiology perspective, IBA are considered to be a common, complex condition with multiple risk factors including advanced age, ancestry, sex (women affected more often than men), smoking, longstanding hypertension, and family history (Brown et al. 2008; The International Study of Unruptured Intracranial Aneurysms Investigators 1998; Juvela et al. 2001; Leblanc 1996). Familial intracranial aneurysms (FIA), a hereditary subtype of IBA, is suspected when two or more affected first- to third- degree relatives are present in a family (Fig. 1a and b) (Bederson et al. 2000). Differences in study populations and methodology have contributed to variability in the reported prevalence of FIA. For example, the rate of detection of intracranial aneurysms when screening first-degree relatives with at least two affected family members has been reported to be 9.2–9.8 % in patients older than 30 years, which is approximately 2–3 times higher than the risk within the general population (Ronkainen et al. 1998; 1997). A more recent study screened for aneurysms among asymptomatic first-degree relatives of families with two affected first-degree relatives *or* three affected second- to third- degree relatives, and found detection rates as high as 20.6 % among patients older than 30 years (Broderick et al. 2009). Individuals with FIA seem to have a more severe phenotype: they are more likely to develop more than one brain aneurysm (Brown and Broderick 2014; Rinkel et al. 1998), and have 17 times greater risk of rupture compared to those with sporadic IBA (Broderick et al. 2009). Furthermore, aneurysms that do rupture tend to rupture at a younger age and at a smaller diameter among patients with FIA (Bacigaluppi et al. 2013; Broderick et al. 2009; Lozano and Leblanc 1987). Along with the increased risk for aneurysm formation and rupture, patients with FIA appear to have a poorer outcome after rupture (Bromberg et al. 1995; Kojima et al. 1998). Generally, screening with magnetic resonance angiography (MRA) or computerized tomographic angiography (CTA) is recommended for individuals with two or more first-degree relatives diagnosed with intracranial aneurysms (Bor et al. 2010, 2014; Brisman et al. 2006; Brown et al. 2008; Ronkainen et al. 1998; Thompson et al. 2015). In the absence of prospective longitudinal studies, expert consensus appears to be that relatives at sufficient risk to merit screening should begin such screening 10 years prior to the earliest age-at-diagnosis in their pedigree.

For the benefit of genetic counselors, who may be consulted by patients concerned about their risk for intracranial aneurysms, or who may discover a family history of intracranial aneurysms during a routine session, we present here a review of the genetics of IBA, and of the known Mendelian syndromes that confer increased risk for IBA. For a review of the natural history of IBA and options for treatment, we refer interested readers to the American Heart Association/American Stroke Association 2015 Guidelines (Thompson et al. 2015) and to a recent review article by Brown and Broderick (2014).

## Family Mapping Studies

Familial mapping studies have been done in large pedigrees that appear to have a single genetic variant as a major risk factor for intracranial aneurysm development. Mapping follows the hypothesis that such a variant will be found in a chromosomal region that has been commonly inherited by all affected family members. Markers of known genomic location, such as single nucleotide polymorphisms (SNPs), are used to derive haplotypes that can in turn be used to identify loci that co-segregate with FIA. Investigations of families and affected sibling-pairs have suggested numerous loci associated with FIA (Table 1). From these studies the loci with the strongest association are 7q11, 19q13, and Xp22. (Farnham et al. 2004; Foroud et al. 2008, 2009; Kim et al. 2011; Mineharu et al. 2007; Nahed et al. 2005; Olson et al. 2002; Onda et al. 2001; Ozturk et al. 2006; Roos et al. 2004; Ruigrok et al. 2008; Santiago-Sim et al. 2009; van der Voet et al. 2004; Verlaan et al. 2006; Yamada et al. 2004). A recent meta-analysis of five familial mapping studies revealed an additional two loci in linkage disequilibrium with FIA, 3q27.3-3qter and 17p12-q21.33 (Biros and Golledge 2008). A SNP association study aimed at replicating loci previously flagged by linkage analysis confirmed the association at 14q23 (found by (Ozturk et al. 2006)) in a cohort of 266 affected and 288 unaffected Japanese individuals (Mineharu et al. 2008). The number of loci identified though linkage analysis indicates there is genetic heterogeneity in FIA.

**Table 1:** Loci associated with familial intracranial aneurysms by linkage analysis

Associated Loci	Study Population(s)	OMIM Locus name	OMIM number	Studied in > 1 population	Study Cohort [affected (unaffected)]	References
1p36.13-p34.3	North American, Dutch	ANIB3	609,122	Yes	12(8) from 1 family 7(10) from 1 family	Nahed et al. (2005) Ruigrok et al. (2008)
2p13	Dutch	-	-	No	7(9) from 1 family^a^	Roos et al. (2004)
4q32.2	FIA Study	-	-	Yes	192 families^b,c^ 333 families^b,c^	Foroud et al. (2008) Foroud et al. (2009)
5p15.2-p14.3	French-Canadian	ANIB4	610,213	No	9(3) from 1 family	Verlaan et al. (2006)
5q22–33	Japanese	-	-	No	104 ASP from 85 families	Onda et al. (2001)
7q11.2	Japanese, North American	ANIB1	105,800	Yes	104 ASP from 85 families 39(0) from 13 families	Onda et al. (2001) Farnham et al. (2004)
8p22	South Korean	ANIB11	614,252	No	9(22) from 5 families	Kim et al. (2011)
11q24-q25	Colombian, North American	ANIB7	612,161	Yes	2 families^b^	Osturk et al. (2006)
12p12.3	FIA Study	-	-	Yes	333 families	Foroud et al. (2009)
13q14.12-q21.1	French-Canadian	-	-	No	10(25) from 1 family	Santiago-Sim et al. (2009)
14q22	Japanese	-	-	No	104 ASP from 85 families	Onda et al. (2001)
14q23	Colombian, North American	ANIB8	612,162	Yes	2 families^b^	Ozturk et al. (2006)
17cen	Japanese	-	-	No	93(27) from 29 families	Yamada et al. (2004)
19q13	Finnish, Japanese	ANIB2	608,542	Yes	48 ASP from 22 families 222 ARP from 121 families 93(27) from 29 families 41(0) from 9 families	Olson et al. (2002) van der Voet et al. (2004) Yamada et al. (2004) Mineharu et al. (2007)
Xp22	North American, Japanese, Dutch	ANIB5	330,870	Yes	48 ASP from 22 families 93(27) from 29 families 7(10) from 1 family	Olson et al. (2002) Yamada et al. (2004) Ruigrok et al. (2008)

Study cohort data corresponds with the reference in each horizontal row. *ASP* Affected sib-pair; *ARP* Affected relative pair

^a^Family is consanguineous

^b^The number of affected and unaffected individuals included in the linkage analysis was not available

^c^A total of 1155 affected and 1895 unaffected family members were genotyped in Foroud et al. (2008) and Foroud et al. (2009). Families were enrolled at recruitment sites located in North America, New Zealand, and Australia

## Genome-Wide Association Studies

Genome-wide association studies (GWAS) interrogate the genome for statistically significant associations between SNPs and disease at a population level. Several loci have been associated with sporadic IBA by GWAS, primarily using large discovery and replication cohorts from the Dutch, Finnish, and Japanese populations (Table 2) (Abrantes et al. 2015; Bilguvar et al. 2008; Deka et al. 2010; Foroud et al. 2012, 2014; Kurki et al. 2014; Low et al. 2012; Yasuno et al. 2011, 2010). The most frequently replicated locus is 9p21.3, which contains the long non-coding RNA, *CDKN2B–AS1*, and is adjacent to the cyclin-dependent kinase inhibitor genes, *CDKN2A* and *CDKN2B*. The same linkage block in 9p21.3 associated with IBA has also been associated with other vascular diseases, including coronary artery disease, myocardial infarction, and abdominal aortic aneurysms: this suggests that there may be a single locus that predisposes to all of these conditions via a common pathology (Helgadottir et al. 2008, 2007). Alg et al. (2013) conducted a meta-analysis of 61 GWAS studies that replicated the association between three loci (9p21.3, 8q11, and 4q31.23) and IBA. In contrast to the postulated high-penetrance Mendelian loci in FIA, loci discerned through GWAS each have a relatively small effect size on the risk of developing IBA. For example, SNPs at the strongest associated locus, 9p21.3, have reported odds ratios between 1.29–1.34 in the major GWAS studies (Bilguvar et al. 2008; Foroud et al. 2014; Yasuno et al. 2010).

**Table 2:** Loci associated with intracranial berry aneurysms through genome-wide association studies

Associated Loci	Study Population(s)	OMIM Locus name	OMIM number	Replicated in > 1 population	Size(s) of Study Cohort	References
2q33.1	Dutch, Finnish, Japanese	ANIB9	612,586	Yes	2196 cases; 8085 controls	Bilguvar et al. (2008)
4q31.22	Japanese	-	-	No	2431 cases; 12,696 controls	Low et al. (2012)
4q31.23	Dutch, Finnish, Japanese	-	-	Yes	5891 cases; 14,181 controls	Yasuno et al. (2010) Yasuno et al. (2011)
5q31.3	Finnish, Dutch	-	-	Yes	2335 cases; 9565 controls	Kurki et al. (2014)
8q11.12–12.1	Dutch, Finnish Japanese	ANIB10	612,587	Yes	2196 cases; 8085 controls 5891 cases; 14,181 controls 406 cases; 392 controls 1483 cases; 1683 controls	Bilguvar et al. (2008) Yasuno et al. (2010) Deka et al. (2010) Foroud et al. (2012)
8q21.3	Dutch, Finnish, Japanese	-	-	Yes	5891 cases; 14,181 controls	Yasuno et al. (2010)
9p21.3	Dutch, Finnish, Japanese, Portuguese	ANIB6	611,892	Yes	2196 cases; 8085 controls 5891 cases; 14,181 controls 406 cases; 392 controls 1483 cases; 1683 controls 4133 cases; 7869 controls 2431 cases; 12,696 controls 200 cases; 499 controls	Bilguvar et al. (2008) Yasuno et al. (2010) Deka et al. (2010) Foroud et al. (2012) Foroud et al. (2014) Low et al. (2012) Abrantes et al. (2015)
10q24.32	Dutch, Finnish, Japanese	-	-	Yes	5891 cases; 14,181 controls	Yasuno et al. (2010)
12q22	Dutch, Finnish Japanese	-	-	Yes	5891 cases; 14,181 controls	Yasuno et al. (2011)
13q13.1	Dutch, Finnish Japanese	-	-	Yes	5891 cases; 14,181 controls	Yasuno et al. (2010)
18q11.2	Dutch, Finnish, Japanese	-	-	Yes	5891 cases; 14,181 controls	Yasuno et al. (2010)
20p12.1	Dutch, Finnish, Japanese	-	-	Yes	5891 cases; 14,181 controls	Yasuno et al. (2011)

The size of each study cohort corresponds with the reference in the same horizontal row. Yasuno et al. (2010) and Yasuno et al. (2011) analyzed the same discovery and replication cohorts, which were expanded on from the cohort studied in Bilguvar et al. (2008)

## Next-Generation Sequencing Studies

Unlike GWAS, but similar to linkage analysis, whole-exome sequencing (WES) aims to identify rare variants that have a large effect size and impart a high risk of developing intracranial aneurysms. Recently, three WES studies of multiplex families diagnosed with FIA have revealed new candidate genes in the development of intracranial aneurysms. All three studies excluded individuals who were diagnosed with a syndromic form of IBA.

The Familial Intracranial Aneurysm (FIA) Study published two exome sequencing studies on seven multiplex families of European-American ancestry. These families were selected for having high numbers of affected individuals, and for having pedigrees consistent with either autosomal dominant or autosomal recessive inheritance. Initially, WES was carried out on 50 affected and unaffected individuals from these families, and analysis identified 96 candidate genes (Foroud for the FIA Study Investigators 2013). In their second publication (Farlow et al. 2015), the FIA Study researchers analyzed WES data from 36 affected and 9 unaffected family members. Unaffected family members were only included if they were above 45 years of age and had received a negative screen by MRA. It is of course possible that some of the family members who were scored as unaffected may yet develop an IBA in their lifetime. For this analysis, Farlow et al. (2015) employed six biological filters on their WES data to generate a list of 68 candidate variants in 68 genes. These filters examined the variant type (non-synonymous SNVs or exonic and splice site indels), variants with a minor allele frequency (MAF) of <0.01, the segregation within families, and the predicted effect on protein (via CADD score ≥ 10, and predicted damaging by Polyphen2 or SIFT). Five variants, found in *GSTCD, DUSP16, LMBR1L, HAL* and *TSC2,* segregated fully with disease in one family and were present in the affected individuals of a second FIA family. None of the variants found in this study overlapped with any GWAS flagged loci (Farlow et al. 2015). This is presumably because rare, highly-penetrant pathogenic variants that result in a Mendelian inheritance pattern in a multiplex family tend not to spread through to the general population (unless some sort of balancing selection occurs).

Yan et al. (2015) had broader inclusion criteria and sequenced families with three or more affected first- to third- degree relatives. They sequenced 42 affected people from 12 families of Japanese ancestry. After filtering for their presence in all affected family members, minor allele frequency (MAF) <0.05, and a predicted damaging effect to the protein (Polyphen2 and SIFT), WES analysis resulted in 78 candidate variants. Two variants, p.Y193F in *GPR63* and p.R142H in *C10orf122* (*TEX36*), segregated with affected individuals in more than one family. While both of these protein changes are predicted to be deleterious, relatively little is known about the gene function and there was not sufficient evidence to classify either mutation as pathogenic. Yan et al. (2015) then selected ten variants from nine genes, based on functions that were plausibly associated to the pathogenesis of intracranial aneurysms, for Sanger sequencing and replication in two additional Japanese cohorts. The first replication cohort consisted of probands from 24 independent FIA families, and the second replication cohort included 426 individuals diagnosed with sporadic IBA. A variant in *ADAMTS15* was significantly associated with the familial cases in the first replication cohort, while variants in *THBD, IL11RA, PAFAH2,* and *ZNF222* had a slightly increased MAF among the sporadic IBA cases in the second replication cohort compared to the Japanese population.

For rare diseases with characteristic phenotypes, a gene-disease association is established when rare variants are present in the same gene in three independent families. For more common diseases such as IBA, issues such as non-penetrance and phenocopies make additional evidence (such as functional studies) highly desirable in order to lower the false discovery rate. Whereas none of the candidate genes yet flagged through these WES studies have sufficient genetic evidence to prove a causal association, genes containing variants that co-segregate with disease in multiplex families are the best candidates to date, and may be implicated (or discarded) by future next-generation sequencing studies.

## Syndromes Conferring Susceptibility to IBA

### Autosomal Dominant Polycystic Kidney Disease

Apart from family history, diagnosis of autosomal dominant polycystic kidney disease (ADPKD) imparts the highest risk for developing IBA (Table 3). Between 4 % and 17 % of patients with ADPKD will develop IBA, with an equal risk distribution between genders (Chapman et al. 1992; Huston et al. 1993; Niemczyk et al. 2013; Ruggieri et al. 1994; Xu et al. 2010). As seen with non-syndromic IBA, the prevalence of IBA increases with age in this disease (Niemczyk et al. 2013; Xu et al. 2010). The combined risk for aneurysmal formation in patients diagnosed with ADPKD and a family history of IBA or SAH increases to approximately 22–25 % (Huston et al. 1993; Ruggieri et al. 1994). Screening for IBA in ADPKD is recommended in patients above 30 years of age, or in patients with a family history of IBA (Xu et al. 2010). Hypertension, found commonly in ADPKD patients, is not considered to be an obligatory risk factor for aneurysm formation in this context. Individuals with ADPKD who have well-controlled hypertension, or hypertension within a normal range, have still been seen to develop intracranial aneurysms (Chauveau et al. 1994; Gieteling and Rinkel 2003; Niemczyk et al. 2013; Xu et al. 2010). Pathogenic variants in the *PKD1* and *PKD2* genes are causative for ADPKD and account for 85 % and 15 % of diagnoses, respectively (Niemczyk, 2015). Patients with pathogenic variants in *PKD1* or *PKD2* appear to have an equivalent risk of IBA (Rossetti et al. 2003). *PKD1* and *PKD2* mutations cause defects in the mechanosensory cilia found on renal and vascular endothelium, which leads to cyst and aneurysm formation, respectively (AbouAlaiwi et al. 2014, 2009; Nauli et al. 2003, 2008). Patients with tuberous sclerosis complex can also have a concurrent diagnosis of ADPKD when their disease is caused by a contiguous gene deletion affecting *TSC2* and *PKD1* at chromosome 16p13.3. These patients not only have more severe kidney disease, but are also at risk for intracranial aneurysms (Chen et al. 2002; Longa et al. 1997; Sampson et al. 1997).

**Table 3:** Prevalence of intracranial aneurysms in selected syndromes and their associated gene(s)

Relative Prevalence	Syndrome	OMIM Number(s)	Prevalence of patients with IBA	Associated Genes
Frequent	Autosomal Dominant Polycystic Kidney Disease	173,900 613,095	4–17 %	*PKD1, PKD2*
Infrequent	Vascular Ehlers-Danlos Syndrome	130,050	12%^a^	*COL3A1*
	Loeys-Dietz Syndrome	609,192 610,168	11–28 %^a^	*TGFBR1, TFGBR2, SMAD3* ^b^
	Marfan Syndrome	154,700	14%^a^	*FBN1*
	Neurofibromatosis Type I	162,200	9–11 %^a^	*NF1*
Rare ^c^	Pseudoxanthoma Elasticum	264,800	-	*ABCC6*
	Hereditary Hemorrhagic Telangiectasia	187,300	~10%^d^	*ENG*
	Multiple Endocrine Neoplasia Type I	131,100	-	*MEN1*

^a^Prevalence estimates may be influenced by selection bias and the inclusion of both fusiform and berry aneurysms

^b^Pathogenic variants in *TGFB2* and *TGFB3* also account for <5 % of LDS cases

^c^Individual case reports only

^d^As the prevalence of IBA in HHT patients is unknown, the prevalence of arteriovenous malformations, which can also lead to cerebral hemorrhage, has been given

### Ehlers-Danlos Syndrome

Ehlers-Danlos syndrome (EDS) is an autosomal dominant connective tissue disorder. In classical EDS, the majority of patients have pathogenic variants in *COL5A1* and *COL5A2*, while in vascular EDS, previously referred to as EDS Type IV, variants in *COL3A1* are responsible for disease (De Paepe and Malfait 2012). Vascular EDS patients have a high risk of mortality due to vascular fragility that often leads to hemorrhage (De Paepe and Malfait 2012). Intracranial aneurysms have been reported in both classical and non-classical forms of EDS, but relatively speaking it is patients with the vascular subtype of EDS who are at the highest risk for IBA formation (Chen et al. 2013; Kato et al. 2001; Kim et al. 2016; Lummus et al. 2014; Mirza et al. 1979; Oderich et al. 2005; Schievink et al. 1990, 2002). Kim et al. (2016) found intracranial aneurysms in 12 individuals, seven of whom had vascular EDS, from a chart review of 99 EDS patients (mean age 41.7 years) who underwent brain imaging. There is currently no consensus on the clinical utility of screening for intracranial aneurysms among otherwise asymptomatic vascular EDS patients. Results of a positive test on such screening may not be easily actionable, because of the high risks associated with surgical intervention. If screening is desired, a non-invasive approach (such as MRA) should be strongly considered in these patients, in order to avoid further weakening of the vasculature (North et al. 1995; Oderich et al. 2005). Certain tertiary care centers with specialized expertise have reported low rates of complications from endovascular procedures in EDS (Lum et al. 2011a, b), such that screening may be safe for EDS patients receiving care at experienced centers.

### Loeys-Dietz Syndrome

Loeys-Dietz syndrome (LDS) is a connective tissue disorder characterized by severe vascular defects, primarily arterial aneurysms, that can hemorrhage or dissect very early in life (Loeys et al. 2005; Williams et al. 2007). Autosomal dominant mutations in TGF-β pathway genes, most frequently *TGFBR1* and *TGFBR2,* cause LDS. Although LDS is not commonly listed as having an association with IBA, a number of patient cases have reported IBA as a feature (Hughes et al. 2011; Levitt et al. 2012; Loeys et al. 2005, 2006; Rodrigues et al. 2009; Williams et al. 2007). Cerebrovascular bleeding is the third leading cause of death in LDS patients, and intracranial aneurysms have been seen at a frequency ranging between 10 and 28 %. (Kim et al. 2016; Loeys et al. 2006; Rodrigues et al. 2009; Vanakker et al. 2011). Although further studies are needed to assess the clinical utility of screening LDS patients specifically for intracranial aneurysms, surveillance of each part of the vascular tree is currently recommended every two years (MacCarrick et al. 2014).

### Marfan Syndrome

Marfan syndrome is an autosomal dominant connective tissue disorder caused by pathogenic variants in *FBN1*. It is characterized by skeletal, ocular, and cardiovascular malformations with wide phenotypic variability. Aortic aneurysm, dissection, and root enlargement are the most commonly reported vascular defects (Dietz et al. 1991; Pyeritz 2016). IBA have been associated with Marfan syndrome through multiple case reports (Finney et al. 1976; Higashida et al. 1988; Matsuda et al. 1979; Ohtsuki et al. 1984; Hainsworth and Mendelow, 1991; Schievink et al. 1997; Stehbens et al. 1989). Conway et al. (1999) did not find sufficient evidence for an association between intracranial aneurysms and Marfan syndrome upon autopsy of 25 patients. Only one patient autopsied was found to have an intracranial aneurysm, a number that would agree with the general population frequency. However, a recent retrospective chart review of 59 Marfan syndrome patients estimated that 14 % have one or more intracranial aneurysms (Kim et al. 2016). Specific screening for intracranial aneurysms is not routinely recommended in Marfan syndrome patients, though it could be considered in family members affected by Marfan syndrome who also have a first-degree relative affected by IBA, whether or not that family member also has a diagnosis of Marfan syndrome.

### Neurofibromatosis Type I

Neurofibromatosis Type I (NF1) is an autosomal dominant condition caused by pathogenic variants in *NF1*. The primary characteristics of NF1 are café-au-lait spots, iris Lisch nodules, and benign neurofibromas (Conway et al. 2001). Vascular abnormalities are also a recognized characteristic of NF1 and patients under 29 years of age have an increased prevalence of IBA relative to the general population risk (Rasmussen et al. 2001). Intracranial aneurysms have been documented as a cerebrovascular feature of NF1 (Friedman et al. 2002; Jett and Friedman 2010). Retrospective reviews of NFI patient data have reported the prevalence of intracranial aneurysms to be between 9 and 11 %. The youngest reported NF1 patient to have an intracranial aneurysm was 1 year of age. However, routine screening for intracranial aneurysms is not recommended for NF1 patients (Muhonen et al. 1991; Rosser et al. 2005; Schievink et al. 2005; Uranishi et al. 1995; Zhao and Han 1998; Zöller et al. 1995). Conway and coauthors (Conway et al. 2001) did not detect any intracranial aneurysms in their autopsy study of 25 NF1 patients between 3 and 69 years of age.

## Other Syndromes

Multiple Endocrine Neoplasia Type I (Adachi et al. 1993), Pseudoxanthoma Elasticum (Allison et al. 1998; Bock and Schwegler 2008; Munyer and Margulis 1981), and Herediatary Hemorrahagic Telangiectasia (HHT) (Willemse et al. 2000) are often mentioned as being associated with IBA. While all have had individual patient case reports which document the presence of intracranial aneurysms, these data are not as robust as are the data for the syndromes listed above and in Table 3. Specific recommendations for IBA screening in these disorders must await larger cohort studies, though in the case of HHT, the currently-recommended screening protocols to detect cerebral arteriovenous malformations (AVM) would be expected to detect IBA as well (McDonald and Pyeritz 2000). Most frequently in HHT patients intracranial aneurysms form along arteries leading into AVM (Willemse et al. 2000). Patients with fibromuscular dysplasia (FMD) are also believed to be at risk for aneurysms in the brain and elsewhere, so current recommendations are that all FMD patients have cross-sectional imaging from head to pelvis with a sensitive method like CTA or MRA (Kadian-Dodov et al. 2016). Though autosomal dominant inheritance has been suggested for fibromuscular dysplasia, definitive genetic candidates have yet to emerge (Kiando et al. 2015). Moyamoya angiopathy, which is also associated with NF1 (Koss et al. 2013), appears to have a pathogenesis that is distinct from that of intracranial berry aneurysms; it is not clear whether the occasional association of intracranial aneurysms with moyamoya (Kawaguchi et al. 1996; Yu et al. 2016) reflects a common pathophysiology at the molecular level, or whether it reflects pathology at one vascular site that perturbs downstream hemodynamics.

## Special Counseling Situations

Certified genetic counselors will already be thoroughly familiar with family trees that suggest Mendelian inheritance that follows autosomal dominant, autosomal recessive or X-linked patterns of inheritance. Even if a DNA-level diagnosis is not available in those situations, counselors will be able to estimate recurrence risks and suggest appropriate follow-up. Where feasible, risk for apparently-unaffected family members may be refined by imaging studies in their living parents or grandparents. Unusual situations may arise that demand consideration of screening protocols that have not been validated by prospective studies. We are aware of at least two independent families in which a child under age 15 suffered a subarachnoid haemorrhage attributed to a berry aneurysm of the cerebral circulation (Fig. 1c and unpublished data). Though neither of these children are known to have another affected first-degree relative, it must be borne in mind that rare children who develop diseases that are otherwise considered common among elderly adults may well have an underlying high-penetrance risk allele. Challenging situations such as these may demand a multidisciplinary approach that includes engagement of the genetic counselor with other medical and surgical practitioners involved in the family’s care. Co**u**nselors may also need to provide an opinion to insurance providers as to the utility of screening in one family member for the purposes of estimating risk in other family members. In the examples given above, screening of clinically-unaffected sibs may be worthwhile, but screening of clinically-unaffected parents may also provide useful information. Were one or more aneurysms detected in a parent, the unaffected sibs would definitely be considered to be at risk and to merit screening under current guidelines.

## Conclusions

Here we have reviewed and summarized the genetic investigations conducted in sporadic and familial intracranial berry aneurysm populations to date. While several loci and candidate genes have been identified, a conclusive gene-disease association has not yet been made for non-syndromic families. Further next-generation sequencing studies are needed to identify a causative gene in families with intracranial aneurysms as the only clinical finding. Patients with FIA are at increased risk for aneurysm formation and rupture compared to the general population, and screening of all first-degree relatives using MRA or CTA is recommended. Definitive identification of a single-gene cause of FIA in a particular family or group of families would allow genetic confirmation as to which family members are at risk. Family members who did not inherit the pathogenic variant would not need to undergo regular screening, and could obtain peace of mind regarding aneurysm formation later in life. As there is not yet a genetic test, or a conclusive familial recurrence rate, genetic counselors will need to estimate patient risk for developing IBA on a case-by-case basis, through analyzing inheritance within a pedigree. Counselors should also investigate the possibility of an associated syndrome by taking a targeted family history; if warranted by specific findings, they could then refer the patient to a medical geneticist or other suitable specialist.

We have also discussed a number of Mendelian syndromes, primarily hereditary connective tissue disorders, which confer increased risk for IBA development. Of these syndromes, ADPKD has the strongest documented association with IBA. The prevalence estimates of intracranial aneurysms in Marfan syndrome, NF1, EDS, and LDS are likely biased to report a higher percentage of affected patients. In the studies discussed, patients identified as being at risk for vascular complications received brain imaging, whereas patients lacking these risk factors did not receive screening and were not included in the prevalence calculation. Additionally, many of these studies also include fusiform aneurysms when documenting the presence of intracranial aneurysms. These two methodological limitations have prevented an accurate measurement of IBA prevalence in the syndromes listed above, which may or may not be significantly higher than the background population risk. LDS prevalence measurements may not be as affected by these limitations if patients are receiving screening every two years as recommended. It is possible that, as more data is collected on the natural history of this syndrome, LDS will move to a higher risk category for IBA. The heterogeneity of findings on IBA within Marfan syndrome, NF1, EDS, and LDS does not give clear direction to genetic counselors. However, genetic counselors should be aware that patients with these syndromes could be at increased risk for IBA development, and may want to recommend screening for patients who present with additional risk factors such as a specific history of IBA in their family members.
